# Exploring the Lived Experience on Recovery from Major Depressive Disorder (MDD) among Women Survivors and Five CHIME Concepts: A Qualitative Study

**DOI:** 10.3390/bs13020151

**Published:** 2023-02-09

**Authors:** Afifah Idris, Noremy Md Akhir, Mohd Suhaimi Mohamad, Norulhuda Sarnon

**Affiliations:** Centre for Research in Psychology and Human Well-Being, Faculty of Social Sciences and Humanities, National University of Malaysia, Bangi 43600, Selangor, Malaysia

**Keywords:** lived experience, MDD, survivor, CHIME framework, women, expert by experience

## Abstract

Objective: Depression is estimated to be the leading cause of disease by 2030 and is already the leading cause in women worldwide. In Malaysia, nearly 2.3 million people have experienced depression at least once. Yet, this problem has not been thoroughly investigated and addressed. Thus, a study exploring the lived experience of the survivors needs to be carried out. With most Major Depressive Disorder (MDD) patients being women compared to men, this study focused on women MDD survivors to understand their journey to recovery. Survivors or also called ‘People with Lived Experience’ (PWLE) have a range of first-hand experiences with treatment and recovery, making them an expert by experience. Method: A qualitative study was conducted using purposive sampling of four women survivors. This method was able to explore the experience of informants rigorously as it gave flexibility and encouraged discussion between researchers and informants. The data from in-depth interviews conducted were then analyzed using thematic analysis, focusing on the key concepts of CHIME conceptual framework of recovery. Results: This study found four major themes with fifteen subordinate themes: survivor’s efforts, challenges, social support, and hopes. The findings of this study were then integrated with CHIME framework, also known as the guiding philosophy of recovery for mental illness patients. Conclusion: These findings contributed to a better understanding of the recovery process and supports needed for MDD patients to recover. In addition, this study also gives hopes that MDD patients can recover, therefore breaking the social stigma still prevalent in the community. Based on these first-hand experiences shared by the survivors, it is hoped that the present interventions conducted by related organizations and caregivers can yield improvements so that the current patients who are still struggling with MDD can recover faster holistically. Limitations and implications for future research have also been discussed.

## 1. Introduction

Major Depressive Disorder (MDD), better known as clinical depression, is a chronic illness that contributes significantly to disease burden [1]. At its worst, depression can lead to suicide. Every year, over 700,000 people die due to suicide, and it is the fourth leading cause of death for people aged 15 to 29 years old [2]. The majority of suicide cases are caused by psychiatric disease, with most important risk factors being depression, substance use disorder, and psychosis [3]. Suicidal thoughts, which are the risk factors of committing suicide, are more common in patients with MDD, with the prevalence among the patients being 48.4% [4]. According to the Diagnostic and Statistical Manual 5 (DSM V), the criteria for MDD are depressed mood, loss of interest and pleasure in almost all activities that have been pleasurable before, significant weight loss/gain, fatigue, feeling worthless or excessive guilt, decreased concentration, and thoughts of death or suicide. These symptoms must persist most of the day, for at least 2 weeks in row. Referring to the National Institute of Mental Health (NIMH), the prevalence of major depressive episodes in 2020 was higher among adult females (10.5%) compared to males (6.2%). The World Health Organization (WHO) estimated that 35% to 50% people from developed countries and 79% to 85% people with severe mental health problems from developing countries were not receiving any proper treatment from the mental health providers [5]. The reasons behind these situations are due to the lack of resources, lack of trained health care providers, and social stigma associated with mental illness [6]. In addition, individuals with depression are often not diagnosed accurately, and individuals who rarely experience depressive disorders have been misdiagnosed and given anti-depressants (WHO, 2021).

In Malaysia, depression has affected nearly 2.3 million people, but this issue has not been fully explored and treated [7]. Referring to the National Health and Morbidity Survey in 2019, about 2.3% or half a million people suffered from depression, with most patients being women (2.6%) compared to men (2.0%). High stress among women occurs due to various responsibilities that need to be performed [8], increasing the likelihood of having depression [9]. Various responsibilities here refer to the situation that is common in urban areas where working women must carry out and adjust tasks at home and office. It has been shown that nearly one-fifth (18.7%) of working women struggle to strike a balance between their roles at home and work [10]. Additionally, some women also play a significant role as informal caregivers because they have to look after the younger members of the family, as well as the elderly and disabled family members [11]. Some are single mothers who are forced to bear greater responsibility after being left by the husband [12]. This condition has indirectly caused a heavy mental burden among them. Other factors that cause high stress and depression among women are stress before and after childbirth (prenatal and postnatal), physical and sexual abuse, and violence committed by the spouse [13]. In one study, it was found that depression symptoms among women might also increase the risk for future intimate partner violence (IPV), and such mental health difficulties possibly making it difficult to prevent or end abusive relationships [14].

When discussing the problems of depression and the actions that need to be taken to hasten the recovery process, the survivors, also called ‘People with Lived Experience’ (PWLE), should be engaged. According to the Mental Health Foundation United Kingdom, apart from PWLE, survivors are also known as ‘mental health service users’ and they are people with mental health difficulties who use mental health services. However, a lot of people who have dealt with mental health issues prefer to use the word ‘survivor’, either to describe their experience as ‘surviving’ mental health issues or ‘surviving’ the use of mental health services. Hence, we decided to use the term ‘survivor’ in this study. Survivors are the experts by experience who have various personal experiences with respect to mental illness, services and rehabilitation, and participation in the design delivery of mental health services [15]. Personal recovery from mental illness has been receiving more attention from policy makers and practitioners (Penas 2020). Therefore, the use of CHIME framework in understanding the personal recovery has been gaining favor. CHIME is the acronym of Connectedness, Hope and Optimism, Identity, Meaning, and Empowerment [16]. The CHIME framework was first designed by Leamy et al. from a systematic review of 87 articles on the framework used for personal recovery in mental health [17]. To date, the framework has provided the most complete picture of recovery process [18].

Due to the high prevalence and morbidity factors, depression has become a main attention for research topic in Malaysia, especially after the COVID-19 outbreak [19]. Previous researchers have extensively addressed the causes and effects of these mental diseases in several studies that have been carried all over the world [20,21]. However, it is difficult to find data on the experiences of survivors among women with MDD, particularly in Malaysia. Therefore, the main aim for this study was to explore the women MDD survivors’ experience throughout their journey to recovery. It is very crucial for everyone to understand how they achieve recovery from their life experiences to help the current patients who are still struggling with MDD. With the increasing numbers of depression cases due to the current COVID-19 pandemic that leads to more emotional pressure especially among women [22], it is hoped that the findings will help to reduce the statistics of MDD in future, as well as the social issues related to depression.

## 2. Methods

### 2.1. Research Design

This was a narrative, qualitative, interview-based study that sought to explore the lived experience of women MDD survivors. The narrative methodology was used in order to appropriately address the study’s objectives since it allowed for in-depth examination of informants’ particular conceptions of recovery as well as the ability to hear the voices of those seeking mental health care. Four women survivors named Mrs. R (31 years old), Miss M (41 years old), Miss S (17 years old), and Mrs. T (30 years old) were selected by using the purposive sampling techniques to undergo an in-depth, one-to-one interview. The search of informants was done through the data provided by the Department of Medical Social Work from Hospital Canselor Tuanku Muhriz and also from the Facebook page called “Depression Survivor Malaysia” group. The first author contacted the potential informants by phone calls and private messaging the informants to search for the inclusion criteria before getting their approval. Twelve potential informants were contacted by the first author, but due to the current history of relapse (mainly due to the COVID-19 pandemic), only four women were selected.

The survivors’ recovery from depression was measured based on the reduction in depressive symptoms that occurred over a relatively long period of time or that showed only mild symptoms. This was in line with the definition of remission by DSM-V. According to DSM-V, a minimum of two months of having no relapse and no significant symptoms can be considered as full remission of depression. Thus, we used this as the baseline for the inclusion criteria of informants. The inclusive criteria for all informants included: (i) having experiences being diagnosed with MDD by psychiatrist; (ii) relying on low-dose medications or having been freed from any medications; (iii) not experiencing relapse for at least two months before the interview; (iv) being aged 55 years and below; and (v) being able to speak and understand Malay or English language. All potential informants were asked to show their book appointments from the hospital or clinic in order to prove that they were qualified to be the informants.

### 2.2. Procedure

Before conducting the interview by using semi-structured questions as a protocol of study, each informant received a briefing about the study’s purpose and the confidentiality of the data. They also had the right to withdraw at any time if they no longer wanted to continue with the research. Written informed consent was obtained from all informants before conducting the interview. All informants also gave their consent to the researchers to publish the data in any publications. The interview was done by the first author in eight sessions (two sessions with each informant with approximately one hour each session) which took place at the informant’s house and by video call. The interview process was done between November 2020 and January 2021. The total duration of interviews with Mrs. S was 3 h, Miss M was 3 h, Miss S was 2 h, and Mrs. T was 2 h. The first author followed the interview guidelines in order to make the informants feel more comfortable during the whole interview process, such as informal conversational interview and standardized open-ended questions. The interviews were started by a question regarding their current health conditions before going into more specific questions on their lived experiences, such as how long they had been diagnosed with MDD, and the factors, struggle, coping skills and social interactions before and after recovering from MDD. For example, the author once asked about the difficulties that they had to face, and all of them answered “stigma”. Then, the author used probing questions to encourage deep thought about specific issue. For example, “what type of stigma have you experienced?” and “how does the stigma affect your journey to recovery?”.

**Figure 1:**
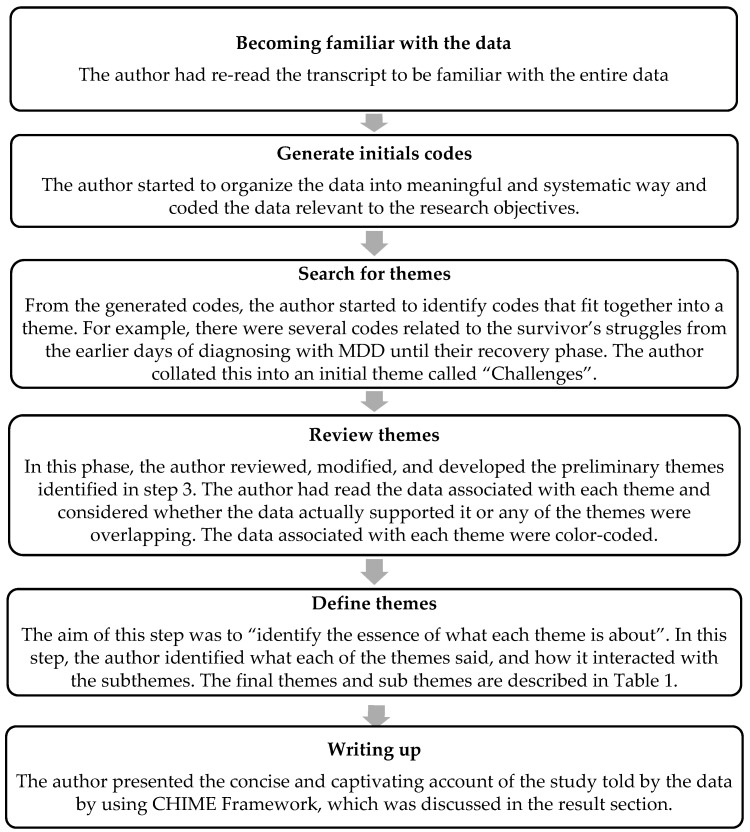
Data analysis process adapted from Braun and Clarke (2006).

The interviews were ended once the data were adequate, and no new themes emerged. The recordings were then transcribed into verbatim text and the researcher identified questions that contained sensitive elements so that the same questions would not be repeated in the next interviews. The transcribed data were analyzed by using the six steps of thematic approach described by Braun and Clarke [23] (see Figure 1). The six steps are as follows (done by the first author):

The first author presented the themes and sub themes of the data to the other three authors, as they were the qualitative experts. Discussion was conducted to determine which themes and sub themes answered the objectives of the study (see Table 1). We discarded codes that were vague or not relevant to the study. Referring to Table 1, those themes and sub themes were the most often spoken by the informants. Only one sub theme, which was the ‘challenges to start a new life’, was shared by one informant, but we decided to include it into the themes as we found that it was a unique experience and would be beneficial for the readers to understand their struggle.

## 3. Results

### 3.1. Demography of Informants

This study involved four women survivors who had been diagnosed with MDD between 3 and 17 years named as Mrs. R, Mrs. T, Miss M, and Miss S. The factors of the MDD occurrences were different from each other. For Mrs. R, the factors were due to psychological and social factors, including the lack of attention from her parents since childhood, often being differentiated with her siblings, and abusive relationships; and a biological factor, which was genetics. She was diagnosed with MDD for over 9 years ago. Similar factors happened to Miss M. For Miss M, who had been diagnosed with MDD over 17 years ago, the difficulty to accept the death of her parents, genetics, and educational stress were the cause of her suffering from MDD. Miss S, who was the youngest survivor in this study, suffered from MDD due to the social aspect which was due to bullying and often being embarrassed by the teachers at school. Miss S was diagnosed with MDD over 3 years ago. As for Mrs. T, she was not sure of the main cause for her MDD that was diagnosed 5 years ago because she had no genetic factors or history of trauma. We believed it was due to the biological factor. One common similarity among all informants was that all of them had a good relationship with their family, be it their spouse, parents, or siblings, becoming their source of social support throughout the journey to recovery. All of the informants had no relapse episode for almost one year before the interviews were done.

The present study revealed four major themes with 15 subordinate themes that highlighted the lived experiences of the women MDD survivors. These major and subordinate themes are discussed as follows (Table 1).

The informants shared the experiences and narratives about their lived experience with MDD related to connection with their caregivers and others in terms of having assistance and social support throughout their recovery journey; hope and feeling of optimism in their future; identity from which the survivors found strength and capability, such as completing task one by one that helped them to recover after the relapse episodes; processes that they went through to regain a more meaningful life; and empowerment that helped them control their life despite all the challenges. Based on these findings, we integrated the data with the CHIME recovery framework into the discussion. Note that the informants all gave consent regarding any of the information.

(a) Connectedness

Connectedness or having contact with the outside world is very important for mentally ill patients to recover. However, to be connected while struggling with depression is not an easy process for the patients. The majority of the survivors asserted that their depression was related to the feeling of isolation and loneliness. In this study, all of the informants were lucky enough to get support from their caregivers. This situation was related to the study by Richardson and Barkham [24], in which caregiver’s social support was crucial for the patient’s ability to recover from mental illness. All of the informants have been receiving full social support from their caregivers, making them not feel isolated and proving that they were loved by the family. The survivors have been doing activities together with their caregivers, such as gardening, sewing, sports, and religious activities. Having a good relationship with family is very important because family is a source of material and emotional support to mental illness patients [25].

*i*. *Social support*

According to the survivors, their recovery process depended on several aspects, namely good care by the caregiver, support from other family members, and indirect assistance from others.

(i) Good care by the caregiver

For Mrs. R, she was very fortunate to get excellent care from her husband. According to Mrs. R, despite having been beaten before, her husband still took a good care of his wife. In fact, her husband never once abused Mrs. R throughout their marriage, especially when Mrs. R’s mental health deteriorated. According to her:

*After I gave birth to my first child, I had post-partum depression. I could feel that I did not like my baby. I felt like it’s hard to take care of the baby. I used to rage, I hit my husband, I kicked him. That’s not normal right? Usually, I raged when my parents were asleep. So, my husband was the one who saw my true colors.*

According to Mrs. R, she was once asked by a psychiatrist to be hospitalized while going through a severe relapse episode. However, on her husband’s assurance, the doctor allowed Mrs. R to go home and be cared for by her husband. According to her:

*I should have been hospitalized. But, because the bed was full, and I had a baby to breastfeed, on my husband’s assurance, I took a very high dose of medicine, and I was like a living corpse for two weeks at home. I just slept all the time. During that time, my husband was a very tired person. Before he went to work, he would prepare the breakfast. At 10 o’clock in the morning, he would come back to bathe the kids and go back to work. At 12 o’clock, he would buy lunch and send to us. At 5 o’clock, he would buy food for dinner, bathe and take care of the kids. I just lied down. Slept.*

As for Miss M, she also received a good care from her brother and his family after they found out about Miss M’s poor health. Miss M also shared how she was taken care of despite being hospitalized in the psychiatric ward. Her brother would commute to the hospital almost every day to feed and ensure that the care by the hospital to Miss M was done well. According to Miss M:

*Although I have been hospitalized, my brother would come every day. He would come to bring the food, and I would eat with him with his wife and his children. That was his routine every day. Only then, he could sleep after seeing me. He also gave some conditions to the ward if they wanted to detain me. He told the doctor and nurses to bathe and feed me every day, just like how he would take care of me at home.*

(ii) Support from other family members

Mrs. R was also lucky to have the support from her eldest sister, especially after learning about her MDD. According to Mrs. R:

*My brother, he’s more silent. Because yeah, man… he doesn’t have many things to say. So, when we meet, we chat as usual. But my sister, maybe because… I am the only sister, when those things went viral (Mrs. R’s sharing about her illness on Facebook), my sister was so shocked, but she did not condemn*.

Since that day, her sister has helped Mrs. R a lot in managing her daily life, especially in her business.

*So since then, she has given a lot of support. I started selling biscuits. At that time, I was pregnant and living on the 4th floor of apartment. My sister will help me pick up the biscuits, take order from her officemate, and help me with sales. Not only that, when I did an online class (teach photography), she would be in that group. She would ask a lot even though she knew the answers already.*

For Mrs. R, her sister’s involvement in her photography class made her feel loved and cared for. Her sister has provided a lot of emotional and spiritual support.

On the other hand, Mrs. T’s parents took the initiative to do Islamic medical treatment. Even though it put some pressure on Mrs. T, seeing her mother’s efforts, Mrs. T complied with her mother’s request. According to Mrs. T:

*There were many places that we went. It’s a bit tense for me. Because when we met the ustaz (Islamic named for Muslim faith healer), he would judge me and tell me to stop taking the medicine. So, that added more pressure.*

(iii) Indirect assistance from others

Miss M also underwent spiritual treatments as one of the recovery processes from depression. She has been involved with this support group since 2018. According to Miss M, her friend she met through this support group helped her a lot and most importantly, understood when she was under stress. According to Miss M:

*There was one time when I shared a room with Mrs. W (her friend from the support group). Mrs. W has already slept that night. But, I couldn’t. I cried and sobbed. It was 3–4 o’clock in the morning. Mrs. W then realized that I was crying. She then asked, “M, why are you crying? Are you sick?”. Then I said, “No. I’m ok”. Then, Mrs. W asked, “Is there anything you want to share with me?” Those questions were like a medicine for me. I felt relieved.*

As for Miss S, when she was at school, the school counselor helped her a lot, especially when she felt uncomfortable in class, and she could feel that there was something that would trigger her illness. According to Miss S:

*Sometimes, if I felt uncomfortable in class, I would go to the counselling room. She (Counsellor) would tell my teacher about my conditions. She explained to the teacher that I couldn’t study like other students.*

According to Miss S, her counsellor would text her mother to ask about her condition. She said:

*The counsellor sometimes messaged my mom to ask about me.*

(b) Hope and optimism

Hope and optimism were very prominent throughout the interviews. All of the survivors expressed their hope that they would fully recover, and that the dose of medication could be reduced. In addition, they really hoped that the stigma could also be curbed. This finding was in line with a systematic review which reported that individuals who thought about their future would strive to achieve their dream based on two orientations: motivation (cognitive desire) and planning (ability to plan) [26]. As Bird et al. [27] showed, hope was an important domain for most individuals, especially during the early stages of recovery, where at this point, there was an increase in hope for many mental illness patients after going through a phase of despair [27]. Hope, according to Favale et al. [28], can be seen as the beginning of the healing process, and it provides a crucial foundation to accomplish a goal.

*ii*. *Hope*

When discussing the issues related to mental illness, the survivors expressed their expectations for self, family, community, and healthcare workers and service providers.

(i) Expectation to self

For Mrs. R, being able to speak in front of many people was a hope placed on her. According to her:

*My hope is one day I could speak in front of many people like in Ted Talk. I have that vision. But I realized, my biggest anxiety right now is speaking English. I can speak English, but with broken grammar.*

According to Mrs. R, when she was able to speak in front of many people, it was satisfying to her.

*I hope I can go to the international stage, not only talking about awareness, but also serving the community since it is my life satisfaction.*

She then added:

*And my dream is, one day I can live just like a normal human being and benefit others. Hopefully, when I die later, there is something I can leave behind. Wow, how big is that dream! (laugh).*

As for Miss S, she hoped that one day she would be able to socialize with friends, just like the old days when she was not diagnosed with MDD. According to her:

*I want to socialize with others. Making new friends. For now, I feel stress whenever I need to go out, meeting other people. I hope I can overcome this in future.*

(ii) Expectations of family

Miss M placed some hope for her family. She wanted her family members to have the same feelings as her, which was to be grateful for the test (illness) that God gave her. According to Miss M:

*Before this, there were those who felt the stress of taking care of me. But, of all their sacrifices, I hope they will feel blessed, just like how I feel. I hope by taking care of me, they know that God has taught them something. “There have been many favors and lessons I got by taking care of my sister.”*

As for Mrs. R, she had an experience being stigmatized by her own in-laws. According to her:

*I hope one day they will understand my illness. It was not that I was lazy to do certain things when we gathered at my in-laws. But sometimes, there was something that triggered me. I could sense that my mental health would be affected. So, I decided to stay inside the room to calm my emotions. I hope one day my in-laws will better understand the symptoms of MDD.*

(iii) Expectations of the community

According to Miss M, the stigma occurred because there was a lack of empathy in society. She hoped that the society would put an effort to understand about mental illness.

*The stigma from people who think that they are healthy, rich, and knowledgeable. Hence, these people are lacking empathy.*

Like others, Mrs. T also hoped that the stigma in society about the patients and mental illness could be reduced or removed. This is because, according to her:

*It is the stigma that actually worsens the patient’s conditions.*

(iv) Expectations of healthcare workers and service providers

Mrs. R hoped for more therapy sessions for mental illness patients. For her, the therapy was very helpful during the recovery process. Unfortunately, not many therapists were available at this point. According to Mrs. R:

*I think the role of psychology students or other people who have the skills in these things are to do the therapy sessions, where patients can come and enjoy the therapy. For example, I have seen a counsellor who does art therapy. Patients will go there and do the art. And the counsellor will then try to tell the story from the art. For example, if the patients use a lot of dark color, maybe they are currently depressed about something.*

The same thing was also voiced by Miss M. She also agreed that therapy was one of the ways to recover from mental illness. But according to her point of view, she needed more spiritual types of therapy, especially for Muslims.

*Patients need therapy. Examples in terms of Qur’anic verses.*

(c) Identity

The creation of an identity to find the strength and capability in reaching personal goals is intimately linked to the recovery from mental illness [29]. For most people, redefining or restoring good thoughts about oneself is the key to healing. Additionally, it is believed that the recovery process involves the discovery of a “new self” or some other sorts of identity modification [27]. According to a study by Apostolopoulou et al. [30], the respondents found that their sense of identity and self-worth were strengthened by their return to control over their lives, the assumptions about responsibility, the perception that they were contributing members of society, the restoration of normalcy, and the restoration of employment. This was in accordance with the current study. The survivors have begun being active in the community. Although at first, they did not believe in their abilities, they managed to prove that they were capable of performing the given tasks. Even though it might appear as a small success by most people, for them, it was a big and worthwhile success, especially after recovering from MDD. Even if the idea of identity is thought to overlap with other recovery concepts, it is inextricably linked to the healing process [31].

*iii*. *Survivor efforts*

Recovery from MDD depended on the survivor’s own efforts. Among the efforts by the survivors were getting help from health services, completing tasks one by one, and taking care of food intake, sleep, and emotions.

(i) Help from health services

Apart from having good coping skills, the recovery process was also aided by the help from health services. When Mrs. R was initially diagnosed with MDD, she was referred to an Occupational Therapist.

*I had four appointments. First, I was given the DASS test. Then, it was massage therapy. Then, there was aromatherapy. Then, the last time I remember, the therapist told me to lie down, the lights were dimmed, music was played, I heard a sound of waterfall, the sounds of birds… From there I understood how the grounding technique worked. We need to focus on the surrounding, not what is on our mind.*

For Mrs. T, apart from the support from family members and her friends, her recovery depended a lot on the medications. According to her, Mrs. T believed more in the medical aspects to speed up her recovery process.

*Hospital medicines are more helpful than traditional medicines.*

Mrs. T has also sought treatments at four different places for her recovery process. According to her:

*I went to therapy at four different places. Two at government hospitals, one at a private hospital, and another one at university’s hospital.*

Just like Mrs. R, Mrs. T was also taught relaxation techniques, especially when having a panic attack. According to her:

*For example, if we are having panic attack, the therapist taught us to do deep breathing.*

(ii) Completing one task at a time

When the researcher asked Mrs. R regarding the important components needed for depressed patients to recover, she asserted that the most important thing was to learn to ‘do it one by one’. According to her:

*Honestly, if I say it all, people will feel heavy. Like myself, as a patient, if I listen to a talk, and that individual tell me to take care of everything, I feel like… I can’t. Because I once went through a phase of depression that made me feel lazy. So, I learnt to do one by one.*

According to Mrs. R, as a patient suffering from depression, sometimes she found it difficult to get up on a daily basis, let alone to do daily chores. Therefore, she learned to solve something one by one or little by little.

*As a patient who has experienced a phase of feeling lazy to live, my advice is try to settle one by one. Like me, I learn by getting up early. When I could get up in the morning, I learned to set small goals. Then, I learned to cook. At first, I tried to cook one dish only. Then, I tried to cook rice with fried fish for example. When I saw everyone eats, I felt happy. Only after that I learned to add dishes. So, when I was able to wake up early in the morning, I learned to do other things too.*

Like Mrs. R, Miss M also emphasized on solving one thing at a time in an effort to recover from depression. According to Miss M:

*During the latest relapse, I couldn’t really afford to do anything. It’s just that God gave me the power to do what I liked to do before. For example, I have learned about 99 names of Allah. But I only remember one name (during relapse). So, I recited it the whole time. I couldn’t afford to get up, but it’s ok. I just recited it while lying down.*

(iii) Taking care of the food intakes, sleep and emotions

Mrs. R also stressed the importance of taking care of the daily food intakes and bedtime. In fact, Mrs. R also limited her screen time in an effort to maintain her mental and physical health. According to her:

*You have to take care of the daily nutrition and sleep. People are always taking easy about sleep. Need to keep track of screen time as well.*

The same thing was also voiced by Miss M. According to Miss M, she now had no problem or did not experience bad effects if she forgot to take the medicine. According to her, her recovery factors also depended on her nutritional, emotional, and sleep patterns.

*No problem (if forget to take medicine). You got to take care of your emotions and food intake (nutrition) and have enough sleep. That’s all.*

(d) Meaning

The process by which people: (i) regain a more meaningful life, (ii) comprehend mental illness and the challenges that surround it, (iii) make the transition to spirituality to form a framework of understanding and explanation of their lives and experiences, (iv) seek an active role in society, and (v) strive for the well-being of life is what is meant by the concept of meaning in the CHIME personal recovery framework [31]. The survivor’s participation in activities and hobbies were seen as their driving force in daily life, described as an empowering experience and giving a long-lasting impact, not only while doing those activities. In fact, survivors could use the moment to be more creative and as a “reference point for life in general” [32]. This concept of meaning gave the survivors an experience of extraordinary joy or achievement. This was due to the fact that it enabled the survivors to enter a free zone where they could give lesser attention to negative thoughts or feelings of loss by giving their lives a new purpose [33]. While most depressed patients would use avoidance coping strategies [34] and try to comprehend what was happening to them, it would make them feel emotionally unstable [7]. Therefore, for MDD survivors, reconstructing purpose into life after recovery was very crucial.

(i) Good coping skills

For Mrs. R, she had some coping skills that helped her through her recovery process. The coping skills that helped her the most was by writing about her illness on social media sites, especially on Facebook. Through this, she gained a more meaningful life after openly sharing her journey via her social media platforms. As she started sharing, her family members started to find out about her illness and stories on Facebook. According to Mrs. R:

*Writing had helped me deleting bad memories in my brain. When I did the writings, it seemed to help remove the unwanted things that I did not want to remember, and those things made my brain feel free.*

Despite getting criticism, Mrs. R still continued her sharing.

*I don’t want people to take many years to get help like me. I know that I’m sick, but I denied it. I got married, and had post-partum depression. I’ve beaten my children to the point of wanting to drown my children. I don’t want people to feel the bad things I’ve been through them. That’s why I wrote.*

Like other survivors, Miss S also had her own coping skills when she felt her mental health was compromised. According to her:

*I listen to songs… I clean the house…*

According to Miss S, listening to the songs could help her emotions to recover.

(e) Empowerment

Empowerment refers to the process through which a person reclaims control over his or her life, accepts personal responsibility, and makes investments in or incorporates positive factors into their life [30]. This idea of empowerment emphasizes the capacity for self-care and planning the daily activities. The ability to take care of oneself is very clearly shown in the interviews that have been conducted. According to all survivors, they were unable to manage themselves during relapse. Everything was done by their caregivers. However, after recovering, the survivors were now able to manage themselves despite still being dependent on medication. They were also able to organize daily schedule and the activities they needed to do every day. As has been shared by all the survivors, they had a reason to live and look forward to each day. This situation could be seen in Mrs. R’s situation where every morning, she was able to organize the activities she needed to do that day. If she felt unwell or was able to identify things that could trigger her mental health that day, she would avoid or stay away from approaching or engaging in the activity, as did the other survivors. Upon recovery, they were able to organize the tasks that needed to be done on a daily basis. This is because, they would self-reflect on their mental health state every morning. This situation was discussed by Bird et al. [27] on the importance of focusing on strength when discussing the concept of empowerment.

*iv*. *Challenges*

The survivors also experienced various challenges during the recovery process. Among the challenges that they faced were the acceptance of family members after being diagnosed with MDD, social stigma, struggle against self-stigma, and the challenges of starting a new life.

(i) Acceptance of family members

For Mrs. R, the first challenge she had to go through was the acceptance of her own family members when they found out about her illness. After her first sharing went viral on the social site Facebook, her mother did not want to communicate with her. Mrs. R was also deeply saddened by the words of her father’s family members, who accused her father of having been reckless in carrying out his responsibilities as a father. According to Mrs. R:

*My mother said, when I got an invitation to go live on television and talk about my depression, my father’s side started to talk about me on their WhatsApp group. My mother told me that she had never seen my father look very sad. Not long after that, he got a call from his sibling. They said, “what are you doing with your daughter? You don’t know how to take care of your children.”*

Her sharing on television and Facebook also made the relationship with her mother better although it took some time. According to Mrs. R, her mother has begun to understand her daughter’s illness.

*So, one day my mom came to my house. We both hugged and apologized. And I think the main core that made me depressed was gone. And my health is getting better after that. (crying).*

As for Miss M, she was fortunate to have an understanding brother who took care from the day she was diagnosed with MDD, but not from the other family members. Her family members were more convinced that her illness was the result of *saka* (a mystical related illness believed for generations by most people). According to Miss M:

*For example, my siblings may understand, but not my uncles and aunties. They don’t understand until now. They still believe in the ‘saka’ thing. So, this thing cannot be solved if they still believe in the mystics.*

(ii) Social stigma

The stigma against depression was also still growing. According to Miss M, the common stigma she received was:

*There is no cure for this disease (laugh).*

The problem of stigma also happened to Miss S. According to her:

*People say depression happens because we don’t pray. Many people feel that this thing (depression) does not exist.*

Mrs. T also talked about about the stigma among health care workers that she had gone through before. According to her:

*Like me… I’ve been through these many times… For example, in the previous relapse, I went to XXX clinic. There, the staff asked me, “Are you not praying? If not, why are you feel like committing suicide, right?” So, I feel like the staff themselves need to be educated.*

(iii) Struggle against self-stigma

For Mrs. R, she not only had to face social stigma from the family and society, but also could not escape from the self-stigma that she felt. According to Mrs. R:

*It’s just the hardest when… for me, when I started to feel better and see the rhythm of wanting to be healthy, but then, I felt I was a loser. I spend much time on Facebook (doing sharing), but sometimes I will indirectly judge and differentiate myself from others. As a result, it affects my mental health.*

According to Mrs. R, sometimes she wanted to stop writing on Facebook. However, she did not want all her previous sharing to disappear if she closed her Facebook account. She was also aware of her responsibility to educate the community about mental illness. According to Mrs. R:

*I feel like I want to close (Facebook). But at the same time, I have a responsibility to society. My way of helping and educating is by sharing. If I deactivate my Facebook, all my writing will be lost.*

According to Miss M, she also had self-stigma due to the social stigma she heard.

*The stigma that said we’re crazy. We can’t recover. No one else is sick like me. It’s only me.*

For Miss S, she also had self-stigma against the illness she suffered before she was diagnosed with MDD. According to her:

*I feel like… crazy… I remember I was very sensitive… Apparently what I felt… was a disease…*

(iv) Challenges of starting a new life

In addition to facing criticism from family members and community, Mrs. R’s biggest challenge was to start a new life. According to her, when her mental health showed improvements, she had to face difficulties to continue her life because she was heavily depending on her husband in her daily life when she was sick. According to Mrs. R:

*Frankly speaking, I wanted to go out to work, but I did not know what to do. For example, when I got an invitation to something, it’s actually my husband who took care most of the things. My husband is the one who encourages me, especially in terms of technical support.*

She added that she was afraid to shoulder a responsibility. According to Mrs. R:

*Because I used to be in a phase where I did not want to do anything in life (when relapse), now I’m learning to take responsibility. But I do not dare to do many things at once.*

She then added:

*It was my husband who taught me to look for solutions, not problems. But, it took me three years to finally taught my brain to always think about solutions whenever problems occurred.*

## 4. Discussion—Key Findings

To the best of our knowledge, this is the first study in the Malaysian context that explored the lived experience of MDD survivors, especially during the COVID-19 outbreak. The present study shed light on the survivor’s experience in their journey to recovery, revealing that even with the different factors that contributed to their MDD, the survivors were able to recover from their illness. What is meant by recovery? Prior to the 1980s, the symptom management was the only focus of treatment for those with severe and persistent mental illness (SPMI) [35]. Anthony [36] put forth, “a deeply personal, unique process of transforming one’s attitudes, values, feelings, objectives, abilities, and/or roles... a method of living a meaningful, hopeful, and contributing life despite with limits created by illness” as one of the first definitions of mental health recovery. Anthony noted that people might recover from illnesses without necessarily being cured, and linked recovery from SPMI to recovery from physical sickness and impairment. Hence, the idea that people with severe and persistent mental illnesses can have fulfilling lives while treating the condition is defined as “recovery”. However, due to its ambiguous definition and undefined bounds, which resulted from its complex and subjective nature, it may be difficult to choose the most suitable instruments for its assessment [37].

While there is still no specific definition of recovery, the present study was able to shed light on what it took to define the meaning of recovery from the survivors’ perspectives. In this study, recovery could be understood as the ‘process’ by which the survivors went through to recover, despite the challenges that they must face. On one hand, they have to deal with the symptoms. On the other hand, the survivors also need to cope with the social stigma, hence the dual burden. With the stigma around mental illness still being strong and ubiquitous in the society, the recovery process was not an easy journey for them. The impact of stigma is two-fold. Stigma hinders mental illness recovery and puts a heavy weight on those who are suffering from it [38]. In addition, social stigma may then lead to self-stigma, increased suicide attempts, relapses, and overall mortality [39]. Women survivors in this study had a good educational background, or at least, had the privileges to pursue their study. One of them was even working on her Doctorate thesis at the time. Even so, they struggled with the MDD and stigma associated with them. However, they have the advantage of receiving proper treatment from the hospital.

One study done among women in India found that, because of stigma, familial pressures, and financial constraints, women were less likely than men to seek and receive mental health care [40]. Interpersonal conflict, difficulty of caregiving, domestic abuse, financial instability, unfavorable reproductive events, and widowhood were all identified by women as contributing factors to depression. Another study by Barbosa et al. [41] also stressed the factors that contributed to women’s depression. In this study, due to post-partum depression, the women refused to take care of the child, losing control of themselves, and they began to be aggressive with the baby, and losing control in interactions with other people, including their husband and family members. Because of biological differences in hormone profiles, which influence the risks and symptoms of mental health disorders, the course of those diseases, and recovery, women are more prone than men to experience severe depression and recurrence [42]. Compared to men, women place more importance on finding therapist who truly listens to them [43]. This, too, has been stressed by the women survivors in this study. They spoke about this when the issue of stigma among mental health professionals were discussed.

Nevertheless, these results make an important contribution to the CHIME framework. The study’s findings supported the five basic ideas of the CHIME model as crucial components of the road to recovery for those struggling with mental health issues. The participants spoke of their desires for a sense of connectedness and wished for a speedy recovery and acceptance in community, optimism, hopes, and aspirations for a better life. The women survivors emphasized how they have reclaimed personal control over their lives with their good coping skills, even if they were only able to finish one task at a time. In addition, the survivors also highlighted a sense of optimism for the future. As has been discussed, the recovery from severe mental illness requires hopes. Having hope lays a solid foundation on which to achieve a goal associated with positive thinking in their self, family, community, as well as the service providers.

If previous study highlighted the hopes for ending depression and discontinuing with treatment [18], this present study found a relatively different perspective. The survivors were hoping for better life and more therapies for mental illness patients in future. The therapies were essential for them to start a new life. In Malaysia, not only are the therapies insufficient, but also the number of psychiatrists is inadequate. There were only 410 registered psychiatrists in Malaysia [8]. Because of that, the survivors were hoping for more expertise in this field so that more therapy by the psychiatrists could be offered to the patients. This showed that ‘hope’ can be important among mental illness patients. Despite the different perspectives, the one thing that is common is that it is hoped for them to recover and have a purpose in life. As they move forward, the women survivors also started to develop an identity to find the strength and capability to achieve goals. They began to actively participate in the community and were able to perform tasks given to them. This present study was in accordance with the study by Schon [44], where the women participants valued their illness as positive and negative, rather than only negative values by the men participants. The women participants viewed their illness as an ‘asset that can contribute to something’ [44]. Similarly, it is the illness that made the women survivors to discover their new self, and slowly start to actively be involved in the community and bravely sharing their journey in order to raise awareness about mental health. That journey creates meaning in their life, in which they regain more meaningful life as they have a long-lasting impact after giving their lives a new purpose. The women survivors also may now have the ability to control over their life and focus on their strengths to overcome challenges. The sense of empowerment showed by them proved that having a supportive network was insufficient. Rather, the recovery was highly dependent on them actively striving to recover and transform their life.

However, this study has numerous limitations. First, the data were collected during COVID-19 outbreak. Thus, the data collection process was difficult due to the Movement Control Order (MCO) during that time. It would be very beneficial if the study could be done in person so that the researcher might have conducted participant observation by being involved in the informants’ activities. By this, the researchers may gain detailed descriptions of the survivors’ actions and experiences in the particular context. Second, the sample size could be larger. Due to the COVID-19 outbreak, we only managed to have these four informants. We contacted a number of potential participants, but due to the pandemic, most of them were having relapses. Hence, they were not eligible in the study. Even so, this study was a qualitative study; we did not aim to generalize results. Rather, we sought to advance our knowledge of the phenomenon being studied and provide fresh research topics.

## 5. Conclusions

Despite the challenges from the early days of being diagnosed with MDD, and throughout their journey to recovery, the study gave hopes to the current patients that recovery from MDD could be achieved as the strength of this study depended on its raw data from the survivors’ own perspectives. Based on the lived experience shared by the MDD survivors, the researchers found that recovery was achievable due to their self-effort, with the strong desire to heal becoming the impetus for them to seek for help. It can also be concluded that the survivors indirectly adopted biopsychosocial and spiritual interventions in their journey to recovery. The participants in this study held the belief that recovery from MDD was feasible and acknowledged that it was a unique process to each participant. Malaysia is undoubtedly not an exception as the WHO predicted that depression would be the main cause of illness in a few more years. Thus, it is hoped that these findings would help relevant organizations to improve the current interventions to help those who are still suffering and struggling with MDD so that they can recover faster, and indirectly help to decrease the MDD statistics in future. As Malaysia continues to advance onto a path of rapid economic development and transformation, we need to be more cognizant of the numerous problems that her peoples must overcome. As a consequence, there is an urgent need for the Ministry of Health to take a major action to address the shortage of psychiatrists so that the current patients may receive immediate and proper interventions. As for the issue of stigma, an effective strategy with the potential to change the public opinions is working with the media to increase awareness of mental health concerns and to create the best standards for reporting and portraying mental illness. We believe that there are still a significant number of undetected cases of depression in Malaysia despite the literature’s high prevalence of depression disorder. Thus, future research is required to determine the prevalence of depression in certain subgroups of people, such as children, youths, and their caregivers. Additionally, there is no research on depression in Malaysian males.

## Figures and Tables

**Table 1:** Themes and subordinate themes.

Subordinate Themes	Themes
Good coping skills	
Getting help from health services	
Completing one task at a time	
Taking care of the food intakes, sleep, and emotions	Survivor efforts
Acceptance by family members	
Social stigma	
Struggle against self-stigma	Challenges
Challenges of starting a new life	
Good care by the caregiver	
Support from other family members	Social Support
Indirect assistance from others	
Expectation for self	
Expectation for family	
Expectation for community	
Expectation for health care workers and service providers	Hope

## Data Availability

Data sharing not applicable.
